# Circadian Rhythm Modulation of Microbes During Health and Infection

**DOI:** 10.3389/fmicb.2021.721004

**Published:** 2021-08-27

**Authors:** James Alexander Pearson, Alexander Christopher Voisey, Kathrine Boest-Bjerg, F. Susan Wong, Li Wen

**Affiliations:** ^1^Diabetes Research Group, Division of Infection and Immunity, School of Medicine, Cardiff University, Cardiff, United Kingdom; ^2^Section of Endocrinology, Internal Medicine, School of Medicine, Yale University, New Haven, CT, United States

**Keywords:** circadian, bacteria, virus, infection, immunity

## Abstract

Circadian rhythms, referring to 24-h daily oscillations in biological and physiological processes, can significantly regulate host immunity to pathogens, as well as commensals, resulting in altered susceptibility to disease development. Furthermore, vaccination responses to microbes have also shown time-of-day-dependent changes in the magnitude of protective immune responses elicited in the host. Thus, understanding host circadian rhythm effects on both gut bacteria and viruses during infection is important to minimize adverse effects on health and identify optimal times for therapeutic administration to maximize therapeutic success. In this review, we summarize the circadian modulations of gut bacteria, viruses and their interactions, both in health and during infection. We also discuss the importance of chronotherapy (i.e., time-specific therapy) as a plausible therapeutic administration strategy to enhance beneficial therapeutic responses.

## Circadian Rhythms

Circadian rhythms refer to 24-h oscillations of biological or physiological activity, which are found in almost all life on earth (Loudon, 2012; Westwood et al., 2019). In mammals, the timing of these rhythms is determined by a central core located in the suprachiasmatic nucleus (SCN) of the mammalian brain, with additional inputs from peripheral oscillators (circadian modulators located in cells or tissues outside of the SCN). These rhythms are strongly induced by light signals via the retinohypothalamic tract (Meijer et al., 1986, 1992) within the SCN, stimulating neuronal firing and inducing rhythmic neurotransmitter secretion, e.g., glucocorticoids such as vasoactive intestinal polypeptide (Shinohara et al., 1994), which coordinate and regulate peripheral rhythms located in other tissues of the body. This coordination by the SCN enables synchronization of these light-controlled rhythms for maximal host benefit, that includes induction of metabolic pathways in anticipation of dietary intake. Peripheral rhythms themselves are also influenced by other factors including hormones and food intake/nutrient availability (Balsalobre et al., 2000; Damiola et al., 2000). Together, the regulation of these rhythms is vital to maintain good health, as disruptions to these rhythms alter susceptibility to infections, autoimmunity, metabolic diseases, and cancer (Turek et al., 2005; Lee et al., 2010; Gibbs et al., 2014; Edgar et al., 2016; Voigt et al., 2016; Ehlers et al., 2018; Hopwood et al., 2018; Krakowiak and Durrington, 2018).

**FIGURE 1:**
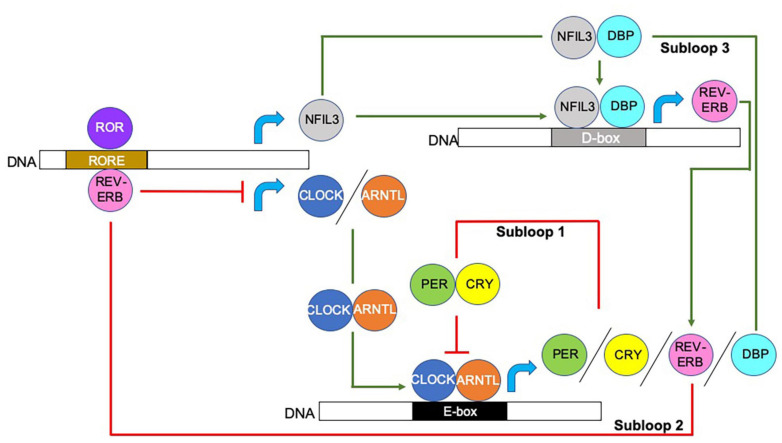
Molecular modulation of circadian rhythm. Circadian rhythms are initiated and regulated by transcriptional/translational feedback loops. Circadian locomotor output cycles kaput (CLOCK) and Aryl hydrocarbon receptor nuclear translocator like (ARNTL; also known as BMAL1) proteins heterodimerize and bind to the E-box motifs of Period (*Per*) and Cryptochrome (*Cry*) genes, driving their transcription (Gekakis et al., 1998; Yoo et al., 2005; Xu et al., 2015). When PER and CRY protein concentrations increase, they too form heterodimers and counter-regulate the CLOCK/ARNTL complex, and thus, inhibit their own expression. CLOCK/ARNTL heterodimer proteins also drive the expression of the nuclear receptors REV-ERBα (encoded by *Nr1d1;* known as nuclear receptor subfamily group D member (Nr1d) 1) and REV-ERBβ (encoded by *Nr1d2*) (as shown by REV-ERB), which bind to retinoic acid-related orphan binding elements (ROREs) located in the CLOCK and ARNTL promoters, repressing *Clock/Arntl* transcription. A third subloop driven by the CLOCK/ARNTL complex, involves the proline and acidic amino acid-rich basic leucine zipper factors, D-box binding protein (DBP), thyrotroph embryonic factor and hepatic leukemia factor proteins (represented as DBP in the figure) (Takahashi, 2017). These proteins interact with the transcriptional repressor Nuclear Factor, Interleukin 3 Regulated (NFIL3; also known as E4BP4), the expression of which is driven by the REV-ERB/ROR loop, at sites containing D-boxes (Takahashi, 2017; Xie et al., 2019). Red lines indicate repression, green arrows indicate activation, blue curved arrows indicate gene transcription. Additional abbreviations used include deoxyribonucleic acid (DNA), Enhancer box (E-box) Destruction box (D-box), retinoic acid-related orphan receptor (ROR).

At the transcriptional level, circadian rhythms are controlled by integrated autoregulatory transcription-translation feedback loops, which direct proteins to bind to sequence-specific elements, resulting in the initiation or repression of circadian gene expression. The interlocking of the three transcriptional feedback loops, shown in Figure 1, generates ∼24-h cycles of transcription with varied phases of expression depending on the proteins available to bind to the promotors or enhancers of target clock-controlled genes (CCGs) (Takahashi, 2017). Together these loops enable a more responsive feedback mechanism that modulates the circadian rhythm in response to environmental changes or stimulations, and these include temperature, hormones, food intake, and microbes.

## Microbes and Health

It has been estimated that ∼1,000 microbial species reside in the human intestines, encoding a vast metagenome with millions of unique genes, modulating host metabolism and regulation of immune responses (Whitman et al., 1998; Qin et al., 2010; Li et al., 2014). Microbial composition can be strongly influenced by many factors including genetics, age, lifestyle and diet (Ley et al., 2006; Turnbaugh et al., 2009; Wu et al., 2011; Goodrich et al., 2014; Bokulich et al., 2016; Odamaki et al., 2016; Sonnenburg and Bäckhed, 2016; Sonnenburg et al., 2016). Crosstalk between bacteria and the immune system is important for the education and development of a mature immune system, including the development of adaptive T-helper 17 cells (Th17) and regulatory T cells (Treg) (Ivanov et al., 2009; Atarashi et al., 2011, 2013), as well as the development of innate lymphoid cells (Gury-BenAri et al., 2016). Importantly, host bacterial composition influences disease susceptibility to obesity (Bäckhed et al., 2004; Turnbaugh et al., 2006; Vrieze et al., 2012), autoimmunity (Frank et al., 2007; Wen et al., 2008; Feng et al., 2010; Lee et al., 2011; de Goffau et al., 2013; Cekanaviciute et al., 2017; Pearson et al., 2019a), cancer (Arthur et al., 2012), and infectious diseases (Dubourg et al., 2017; Kaul et al., 2020; Yeoh et al., 2021). Furthermore, host bacterial composition can modulate responses to immunotherapy (Gopalakrishnan et al., 2018; Routy et al., 2018; Mager et al., 2020) and drugs (Wu et al., 2017; Zimmermann et al., 2019). Taken together, these reciprocal interactions highlight the important role bacteria play in shaping both our immune responses and therapeutic success against a variety of microbial infections, but also other conditions.

Similarly to bacteria, viruses, which are obligate parasites requiring infection of host cells in order to multiply, have co-adapted to their hosts and endogenous viral elements have been incorporated into host genomes millions of years ago (Simmonds et al., 2019). Alongside bacteria, viruses are often associated with many public health outbreaks, e.g., norovirus and rotavirus (Atmar and Estes, 2006; Harris et al., 2008; Lysén et al., 2009; Wikswo and Hall, 2012). Viruses have also been associated with global pandemics including the H1N1 influenza virus (Smith et al., 2009a, b) and SARS-CoV-2 (Wu Y. et al., 2020; Zhu et al., 2020), which have been responsible for the deaths of millions of people worldwide. Thus, there is great need to better understand how antiviral responses are stimulated and how they can be modulated to achieve protective antiviral responses, without promoting significant inflammation and cell damage. Therefore, better understanding of the mechanisms by which microbes can be modulated, including by circadian rhythms, are essential for identifying ways to improve the harnessing of microbes for promoting disease protection and therapeutic success.

### Circadian Oscillations of Commensal Bacteria

**FIGURE 2:**
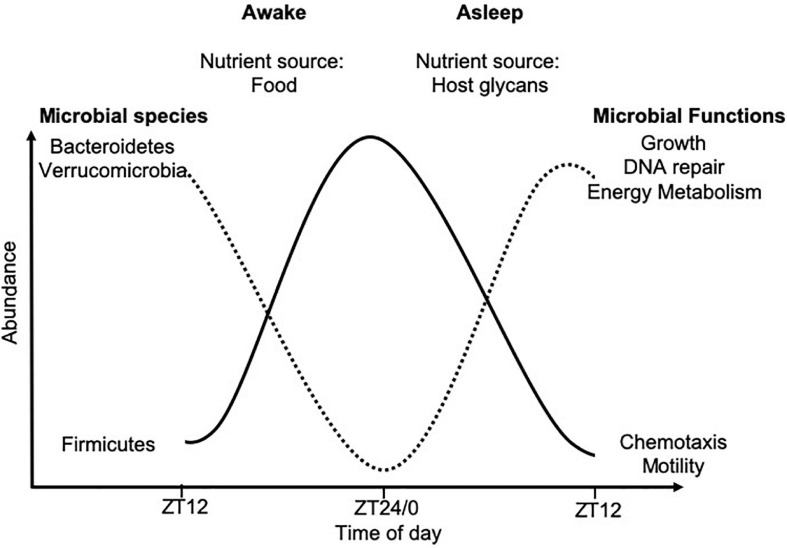
Time-of-day-dependent commensal microbial changes. Oscillations in microbial abundance and functions change at different times-of-day. The abundance of species belonging to the Firmicutes phylum (solid black line) increase in response to dietary intake, while species of the Bacteroidetes and Verrucomicrobia phyla (dotted black line) increase in abundance once all the nutrients from the food have been metabolized, due to their ability to metabolize host glycans. These data were compiled from animal studies with data shown over a 24-h period from ZT12 on 1 day, to ZT12 the following day. ZT12 refers to 12 h post light onset, while ZT24/0 refers to the end of the dark period and the start of the light cycle in standard 12-h light/dark housing conditions, with mice fed *ad libitum*.

Within the intestine, circadian rhythms alter the regeneration of intestinal stem cells, production of gastric acid, gut motility, nutrient absorption and mucosal immunity (Larsen et al., 1991; Pan and Hussain, 2009; Hoogerwerf et al., 2010; Karpowicz et al., 2013; Mukherji et al., 2013; Yu et al., 2013; Wang et al., 2017). In addition, circadian rhythms alter the gut microbial composition and functions (Thaiss et al., 2014, 2016; Zarrinpar et al., 2014; Liang et al., 2015). This microbial rhythmicity is strongly linked to food intake and thus, nutrient availability. Mice housed in standard 12-h light/dark cycles with food *ad libitum*, were found to consume the majority of their food in the dark cycle, when they are most active, corresponding with cyclical changes in the cecal microbial composition, as studied by 16S rRNA sequencing (Hatori et al., 2012; Zarrinpar et al., 2014). Driving these microbial oscillations were time-of-day-dependent changes in the microbial abundances of species belonging to Firmicutes, Bacteroidetes, and Verrucomicrobia (Figure 2). Importantly, these time-of-day-dependent changes in microbial abundance were also associated with functional changes (Thaiss et al., 2014, 2016). Time-restricted feeding protocols, whereby mice are fed either in the light or dark cycles only, confirmed these microbial oscillations were regulated by dietary intake, rather than light exposure (Mukherji et al., 2015a, b).

Diet can also affect microbial rhythms, as mice given standard chow exhibited diurnal oscillations in microbial abundance, whereas, mice fed a high-fat diet showed significantly blunted microbial rhythms, which could be partially restored if given time-restricted feeding (Zarrinpar et al., 2014; Leone et al., 2015). A recent study in humans showed that arrhythmic bacteria (not changed in abundance at different times-of-day) could be linked to susceptibility to developing type 2 diabetes (Reitmeier et al., 2020), which confirmed the importance of these microbial rhythms in human health. It is not clear whether arrhythmic or rhythmic bacteria regulate susceptibility to other diseases. Obesity and obesity-associated health problems, e.g., hypertension and type 2 diabetes, are continuing to increase in prevalence worldwide (Blüher, 2019), and these individuals are more likely to develop severe complications and higher mortality from infections including influenza H1N1 (Huttunen and Syrjänen, 2010; Fezeu et al., 2011; Louie et al., 2011; Milner and Beck, 2012) and more recently SARS-CoV-2 (Sattar et al., 2020; Wu C. et al., 2020; Zhou et al., 2020). Thus, greater numbers of the people within the population are likely to be more susceptible to infectious disease in the future. Therefore, it is important to understand the contribution of the microbial rhythms in mediating susceptibility to infection and whether time-restricted feeding can reduce an individual’s susceptibility to infection.

It should also be noted that some bacteria have their own circadian rhythms. Cyanobacteria, e.g., *Synechococcus elongatus*, encodes three core clock genes (*kaiA*, *kaiB*, and *kaiC*), which regulate different bacterial biology including cell division, photosynthesis, and amino acid uptake, providing a survival advantage to the bacteria (Kondo et al., 1994). *Klebsiella* (previously designated *Enterobacter*) *aerogenes*, a commensal bacterium from the human gastrointestinal system, exhibits rhythms in swarming/motility in response to oscillating host melatonin levels (Paulose and Cassone, 2016; Paulose et al., 2016). This was postulated to be related to the bacteria expressing sequences similar to human melatonin binding sites, although the similarity is only ∼24–42%; thus, further studies are needed (Paulose et al., 2016). This bacterium also modulates circadian rhythms in response to temperature changes (Paulose et al., 2019), which affects host temperature changes after infection, and, in turn, it is likely to influence microbial commensal circadian rhythms. In addition, *Escherichia coli and Pseudomonas aeruginosa* encode receptors for blue and red light, respectively, enabling them to respond to light (Davis et al., 1999; Perlova et al., 2019), a dominant influencer of circadian rhythms; however, it is unknown if these receptors would induce bacterial oscillations in the intestine, given that there is no direct light source. Similarly, *Legionella pneumophilia* encodes homologues of the KaiBC proteins of cyanobacteria that regulate circadian gene expression; however, these gene homologues in *Legionella pneumophilia* appear to only enhance stress resistance (Loza-Correa et al., 2014). Given ∼1,000 microbial species live in the human intestine, it is likely that other bacteria may express genes or responses that can be controlled intrinsically in a circadian manner. More studies are needed to probe whether these intrinsic oscillations have any impact on other microbe rhythmicity or immune functions.

### Host Influences on Bacterial Circadian Oscillations

Host circadian rhythms can also alter bacterial oscillations. Studies using mice deficient in either *Bmal1* or *Per1/2*, or mice with a mutation in the *Clock* gene (altering the period, precision, and persistence of circadian rhythms) revealed that all these mice exhibit altered microbial rhythmicity and composition (Thaiss et al., 2014; Liang et al., 2015; Voigt et al., 2016). It is likely that this is a two-way interaction as germ-free (GF) mice, which lack microbiota, have different intestinal and hepatocyte circadian gene expression when compared to either specific pathogen-free (SPF; which have microbiota) mice or conventionalized mice (GF mice colonized with microbiota from SPF mice) (Leone et al., 2015; Weger et al., 2019). Leone et al. (2015) found that these diurnal oscillations modulated bacterial metabolite production, particularly butyrate, which had direct influence on circadian rhythm gene expression in the hepatocytes. Importantly, diet also modulated bacterial metabolites – butyrate oscillated in normal chow-fed mice, whereas hydrogen sulfide oscillated in high-fat diet fed mice. This further highlights the important link between diet, microbiota, and circadian rhythms.

The initial recognition of bacteria and virus by the host is through pathogen recognition receptors (PRR) including toll-like receptors (TLRs), nod-like receptors (NLRs), and inflammasomes, all of which oscillate in their expression at different times-of-day, in both hematopoietic and non-hematopoietic cells (Silver et al., 2012a, b, 2018; Mukherji et al., 2013; Wang et al., 2017, 2018; Pourcet et al., 2018). The presence of gut microbiota influences both their circadian clock and PRR expression (Mukherji et al., 2013; Wang et al., 2017). Interestingly, the circadian clock in intestinal epithelial cells relies on IL-23 secreted by TLR-stimulated dendritic cells, which activates type 3 innate lymphocyte (ILC3) cells to subsequently secrete IL-22 that in turn activates the circadian rhythms in epithelial cells (Wang et al., 2017). Inflammasomes are also regulated by circadian genes as Rev-erbα represses *nlrp3* transcription by directly binding to the promoter region of the *nlrp3* gene (Wang et al., 2018). While bacterial composition is regulated in a circadian manner, it is likely therefore, that microbial ligands will oscillate and thus responses to bacterial/viral ligands may vary in a time-dependent manner. This is likely to be important, as studies in macrophages have shown that the expression of different TLRs peak at different times (Silver et al., 2012a), suggesting that anti-microbe responses may also change at different times-of-day. Furthermore, these time-of-day-dependent PRR rhythms can also contribute to vaccine responses, as has been shown for B cell-secreted antibody responses (Silver et al., 2012b) (discussed in more detail later). To our knowledge, no studies to date have correlated the microbial oscillations with ligand abundance which could regulate PRR signaling at different times-of-day. This may partially explain the disparity between responses to different microbes at different times-of-day.

Hormones, e.g., glucocorticoids, are known to modulate circadian rhythms (Sollars et al., 2014), including influencing immune cell trafficking (Shimba et al., 2018), and immune responses (Gibbs et al., 2014). Interestingly, gut microbiota is also interconnected with glucocorticoids, as the microbiota can metabolize glucocorticoids, generating steroid metabolites (Morris and Brem, 2019), which affects the host’s immunity and behaviour (Wohlgemuth et al., 2011; Luo et al., 2018), whereas glucocorticoid treatment can alter the gut microbiota composition (Wohlgemuth et al., 2011; Huang et al., 2015; Guo et al., 2020). Thus far, little is known about the crosstalk between circadian rhythms, microbiota and glucocorticoid responses.

Sex has also been shown to influence microbial rhythmicity, with female mice exhibiting greater oscillations in microbial abundance (as determined by 16S copies/g feces), and a possible reduced fecal bacterial load compared to males (Liang et al., 2015). Given that sex can alter the microbial composition and disease susceptibility (Markle et al., 2013; Yurkovetskiy et al., 2013; Ding and Schloss, 2014; Singh and Manning, 2016; Sinha et al., 2019), it is possible that these alterations in microbial rhythmicity may play an important role in contributing to susceptibility to the diseases that affect more women than men, e.g., breast and thyroid cancers and autoimmune diseases such as systemic lupus erythematosus (Rees et al., 2016; Westergaard et al., 2019).

Importantly, changes in time-of-day or disruptions to the circadian rhythms can have large impacts on the host’s susceptibility to a number of health issues including obesity and susceptibility to infections. Bellet et al. (2013); Nguyen et al. (2013); Gibbs et al. (2014), viral pathogens (Edgar et al., 2016; Gagnidze et al., 2016; Ehlers et al., 2018) (discussed next), and parasites (Hopwood et al., 2018), indicating the importance of the host circadian rhythm in susceptibility to disease.

### Circadian Rhythm Influences the Host’s Susceptibility to Pathogens

**FIGURE 3:**
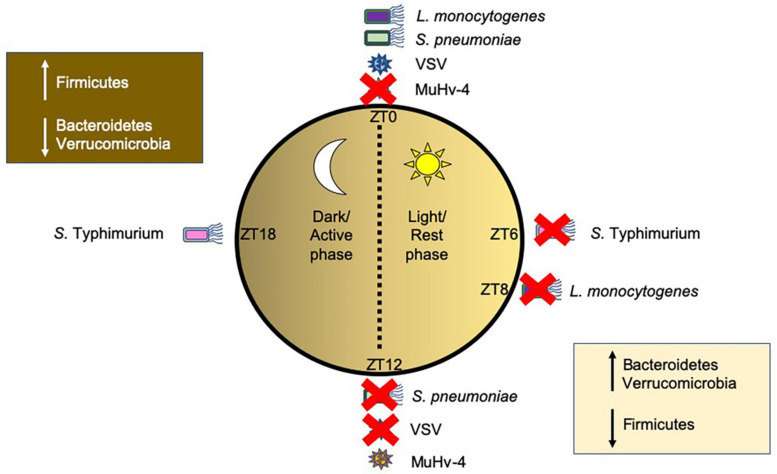
Time-of-day-dependent changes in modulating host susceptibility to pathogens. Host circadian rhythms influence the promotion of bacterial and viral disease depending on the time-of-day that the host was infected. Pathogens are shown around a circle representing different times of day with ZT0 representing the start of the circadian cycle (Bmal1 induction) and rest/light phase and ZT6, ZT8, ZT12, and ZT18 representing 6, 8, 12, and 18 h past light onset, where at ZT12 the dark/active phase starts. Red crosses indicate a time-of-day the host immune response is better prepared to prevent infection, while pathogens without a red cross indicate a time that is better suited for the pathogens to cause disease. This figure was generated from published murine studies. Other pathogens, including human pathogens, are likely to follow similar patterns. Changes in commensal gut microbial species at different times-of-day are also shown in boxes. Abbreviations: ZT, Zeitgeber, *S. pneumoniae* (*Streptococcus pneumoniae*), *S.* Typhimurium *(Salmonella* Typhimurium), *L. monocytogenes* (*Listeria monocytogenes*); VSV, Vesicular stomatitis virus; Mu-HV-4, Murid herpesvirus 4.

Both bacterial and viral pathogens exhibit time-of-day-dependent differences in their ability to replicate or induce immune responses (Figure 3). Time-of-day is often designated by Zeitgeber (ZT) measurements, whereby ZT0 represents the start of the circadian rhythm, i.e., when the light starts; any time after ZT0, for example ZT6, represents the number of hours (6 h) past the initiation of the circadian rhythm. In mice housed in standard 12 h light/dark cycles, ZT0-ZT12 represent the light phase when mice are resting, while ZT12–ZT24 (ZT0) represent the dark phase, when mice are most active. Mice infected with *Salmonella* Typhimurium (Bellet et al., 2013) or *Streptococcus pneumoniae* (Gibbs et al., 2014) showed enhanced ability of the host to reduce pathogen colonization and boost anti-bacterial immunity when infected during the active phase (ZT16 or ZT12, respectively), compared to the rest phase (ZT4 or ZT0, respectively). Similarly, mice infected with *Listeria monocytogenes* (Nguyen et al., 2013) exhibited better resolution of infection when infected later in the rest phase at ZT8, compared with infection at the start of the rest phase at ZT0. Clearly, host circadian rhythms modulate susceptibilities to bacterial pathogens at different times-of-day. Using myeloid cell-specific Bmal1-deficient mice (*Arntl^*LoxP/LoxP*^Lyz2^*Cre*^*) to study diurnal rhythms of inflammatory Ly6C^hi^ monocytes, Nguyen et al. (2013) found that the altered immune responses to *Listeria monocytogenes* infection of the mice correlated with different times-of-day. The *Arntl^*LoxP/LoxP*^Lyz2^*Cre*^* mice developed enhanced bacterial-induced inflammation driven by increased recruitment of inflammatory monocytes and secretion of chemokines (CCL2, CCL8) as well as cytokines (IL-1β and IL-6). In addition, TNFα^+^iNOS^+^ DCs and IFNγ-secreting T cells were also increased. Thus, Bmal1is important in the control of Ly6C^hi^ oscillations, limiting their recruitment at specific times-of-day to prevent inflammation and promote host survival. Similarly, in response to bacterial LPS and *Salmonella* Typhimurium infection, Clock-mutated bone marrow derived monocytes (BMDMs), causing a phase shift of 8 h, exhibited reduced proinflammatory cytokine responses compared to wild-type BMDMs (Bellet et al., 2013). While macrophages and monocytes had been implicated in the circadian responses to both *Salmonella* Typhimurium (Bellet et al., 2013) and *Listeria monocytogenes* (Nguyen et al., 2013), they were not essential in mediating host susceptibility to *Streptococcus pneumoniae* infection at different times-of-day (Gibbs et al., 2014). Instead, bronchial airway epithelial cells were found to modulate host susceptibility to infection mediated through rhythmic glucocorticoid signaling, which drove oscillations in *Cxcl5* expression from the epithelium, and thus differences in recruitment of neutrophils to the site of infection. Both responses to *Salmonella* Typhimurium (Bellet et al., 2013) and *Streptococcus pneumoniae* (Gibbs et al., 2014) involve bacterial ligand (LPS/pneumolysin toxin) binding to TLR4 to trigger an immune response. As mentioned earlier, host TLRs can oscillate at different times of day both in the intestinal epithelium and in immune cell subsets, including macrophages and neutrophils (Silver et al., 2012a, b, 2018; Mukherji et al., 2013; Wang et al., 2017). Thus, it is likely that TLR oscillations are also involved in the regulation of the immune responses to these bacteria. While TLR4 oscillations were not observed in the mouse lung (Gibbs et al., 2014), this does not exclude the role of TLR4 rhythmicity, as a heterogeneous population of immune cells is present in the lung and each cell subset may have altered TLR rhythmicity. Thus, more studies are required to elucidate whether TLR signaling is vital in modulating these circadian differences.

Crosstalk between viruses and host circadian rhythms influences host circadian rhythm modulation of viral infections and reciprocal viral modulation of host circadian rhythms. Unlike bacteria, viruses do not themselves have circadian rhythms of their own but viral infection of the host cell can influence and be influenced by host circadian rhythms. Mice infected with either murid herpesvirus 4 (MuHV-4) (Edgar et al., 2016) or herpes simplex virus 2 (HSV-2) (Matsuzawa et al., 2018) during the rest phase (ZT0–ZT12) showed greater protection from viral infection, compared to the mice infected during the active phase (ZT12–24). This protection correlated with high *Bmal1* gene expression in infected fibroblasts/keratinocytes, which was confirmed by the studies using Bmal1-/- mice, whereby viral load was significantly higher in Bmal1-/- mice compared to wild-type mice. In addition, mice infected with the DNA virus, herpes simplex virus 1 (HSV-1) as well as the RNA viruses, Sendai virus and murine influenza A (IAV) also showed enhanced viral replication *in vivo* in Bmal1-/- hosts (Edgar et al., 2016; Ehlers et al., 2018). Moreover, viral replication of the RNA viruses, respiratory syncytial virus (RSV) and parainfluenza virus type 3 (PIV3) was also increased in Bmal1-deficient cells *in vitro* (Majumdar et al., 2017). It was intriguing that Sendai virus, IAV, RSV, PIV3 (all RNA viruses), which encode their own RNA-dependent RNA polymerases, and therefore do not utilize host transcriptional machinery for viral gene expression, unlike the herpes viruses (DNA viruses) which are more reliant on host transcription for gene expression, yet Bmal1-deficiency increases viral replication in all these viruses. Treatment of human A549 cells (an alveolar carcinoma line) with 100 nM siRNA targeting Bmal1 (72% knockdown) led to higher PIV3 and RSV infections, respectively, agreeing with studies conducted in Bmal1-/- mice (Majumdar et al., 2017). Seasonal variation in *Bmal1* expression in humans, namely *Bmal1* expression is lower in the winter months, coincides with higher disease risk marker expression. This seasonal variation in *Bmal1* may explain the increased susceptibility to viral infections during winter months (Dowell, 2001; Dopico et al., 2015; Martinez, 2018). Protein analysis comparing primary fibroblasts from WT and Bmal1-/- mice indicated that circadian rhythms can control viral replication through many mechanisms, including alteration of proteins related to virus particle uncoating, genome trafficking, chromatin assembly, virus protein biosynthesis, viral assembly and egress (Edgar et al., 2016). In addition, expression of Nectin1, the receptor for HSV-2 (Taylor et al., 2007), oscillates in both mouse skin and human keratinocytes, peaking at ZT18, suggesting a mechanism for the increased infection at ZT18 vs. ZT6 (Matsuzawa et al., 2018). Altered immune responses to viruses have also been implicated in time-of-day-dependent susceptibilities to infection. For example, Sendai virus infections in Bmal1-/- mice exacerbated inflammation, by increasing CCL2, CXCL5, IFNs, and IL-6 production (Ehlers et al., 2018). Furthermore, in studies of RSV infection, human nasal wash samples showed virally-modulated impacts on circadian genes. In infants hospitalized with RSV bronchiolitis, reduced *Bmal1* expression (Ehlers et al., 2018) was found in the nasal wash samples compared to uninfected controls, and this likely contributed to the higher levels of viral replication, and thus, worse clinical manifestation.

Interestingly, some viral infections exploit host susceptibility during the rest phase, suggesting different virus-specific requirements at different times-of-day. Mice intranasally infected with vesicular stomatitis virus (VSV) developed encephalitis, but interestingly, the mice were more protected from the disease if the infection was at ZT12 compared with those infected at ZT0 (40% vs. 95% mortality, respectively) (Gagnidze et al., 2016). Further, the protective effect was due to Rev-Erbα-mediated *ccl2* repression at the start of the active phase, leading to reduced recruitment of inflammatory cells, and thus reduced disease severity. Similarly, Rev-Erbα agonists repressed the replication of hepatitis B and C viruses (HBV, Zhuang et al., 2021; HCV, Zhuang et al., 2019) and human immunodeficiency virus (HIV, Chang et al., 2018; Borrmann et al., 2020), whereas Bmal1 expression promoted viral replication. It is noteworthy that Bmal1 is negatively regulated by Rev-Erbα. It is clear Bmal1 has a pleiotropic role whereby Bmal1 promotes VSV, HBV, HCV, and HIV replication, while suppressing IAV, RSV, HSV-2, PIV3, HSV-1, and MuHV-4 replication. Therefore, there are likely to be additional virus-specific pathways, which modulate circadian rhythms that need to be further elucidated.

Other studies have shown that viral infections disrupt epigenetic mechanisms, affecting the functioning of the circadian clock. Using an *in vitro* HCV infection system, HCV core protein (genotype 1b) decreased PER2 and CRY2 protein levels in infected HuH-7 cells (human hepatoma cell line), whereas overexpression of *Per2* in HuH-7 cells reciprocally decreased HCV RNA replication (Benegiamo et al., 2012). Further study suggested that the CDH1 (E-cadherin) promoter region of the HCV core protein(+) Huh-7 cells becomes substantially hypermethylated, and the reduced CDH1 protein expression in hepatocellular carcinoma patients was associated with poor prognosis (Chen et al., 2014). Hypermethylation of the CDH1 (E-cadherin) promoter region also increased SIRT1 protein levels in HCV core protein+ Huh-7 cells (Ripoli et al., 2011). Importantly, SIRT1 is known to regulate the circadian clock in hepatocytes, controlling regeneration, proliferation, and metabolism of hepatocytes (Bellet et al., 2016; Sato et al., 2017). Thus, HCV modulates circadian rhythms via manipulation of the epigenetic architecture. Further investigation into epigenetic changes induced by viruses and bacteria in modulating circadian rhythms is needed.

It should be noted that the effect of circadian rhythms on the same virus could have different effects, dependent on whether infection is active or latent. Acute herpes viral infections can be influenced by circadian rhythms, whereas there was no evidence suggesting that latent infections are similarly influenced (Edgar et al., 2016). However, whether particular stimuli that induce reactivation of latent viral infections can modify the influence on circadian rhythms remains to be studied. In addition, whether circadian dysregulation promotes viral reactivation also is yet to be studied.

Together, these studies, just discussed, indicate that the time-of-day can substantially alter the potency of the pathogenicity and the host immunity. Importantly, the time at which the host is most susceptible to pathogen infection varies and depends on the type of virus and bacteria, all of which may have important clinical implications. Thus far, circadian rhythms and timed viral co-infection have not been studied; however, this type of investigation may enable us to better understand how circadian rhythm modulated pathways and the pathogens interact. Studying bacterial and viral pathogens known to differentially modulate host susceptibility at different times-of-day may provide important insight into new pathways that could be therapeutically targeted to boost host protection. Alterations in the oscillations of gut commensal microbiota composition have yet to be studied in response to viral and bacterial pathogens, which likely play an important role in modulating host susceptibility to infection, as discussed next.

## Bacterial-Viral Crosstalk in Infection

It is known that viral infections can alter the gut microbiota composition, including HIV, influenza virus, norovirus, rotavirus, HBV and HCV in humans, which can in turn influence disease severity (reviewed in Li et al., 2019; Yuan et al., 2020). Individuals with HIV infection had reduced microbial richness and depleted *Bacteroidia* members but their stool samples were enriched in species belonging to the Proteobacteria phyla (Vujkovic-Cvijin et al., 2013). Interestingly, the reduced alpha diversity (number of different microbial species present) (Noguera-Julian et al., 2016), was associated with the severity of immunodeficiency (Noguera-Julian et al., 2016). However, this could be restored following anti-retroviral therapy (Ji et al., 2018). Importantly, the alterations in microbial composition were also associated with altered microbial functions, whereby HIV infection repressed the generation of proline, phenylalanine, and lysine (all amino acids) (Serrano-Villar et al., 2016), while promoting tryptophan catabolism (Vujkovic-Cvijin et al., 2013) by the microbiota. Given Proteobacteria and Bacteroidetes (which includes the *Bacteroidia* members) oscillate, it is conceivable that circadian rhythms influence the microbiota composition in those individuals. Administration of antibiotics/probiotics/prebiotics in mice, as well as studies in germ-free (GF; which do not have any bacteria) or gnotobiotic mice (which have a defined microbial community), have confirmed that viral-bacterial crosstalk can modulate susceptibility to viral infection and disease, e.g., norovirus infection (Schaffer et al., 1963; Isaak et al., 1988; Cadwell et al., 2010; Ichinohe et al., 2011; Basic et al., 2014; Osborne et al., 2014; Robinson K. M. et al., 2014; Pearson et al., 2019b).

**FIGURE 4:**
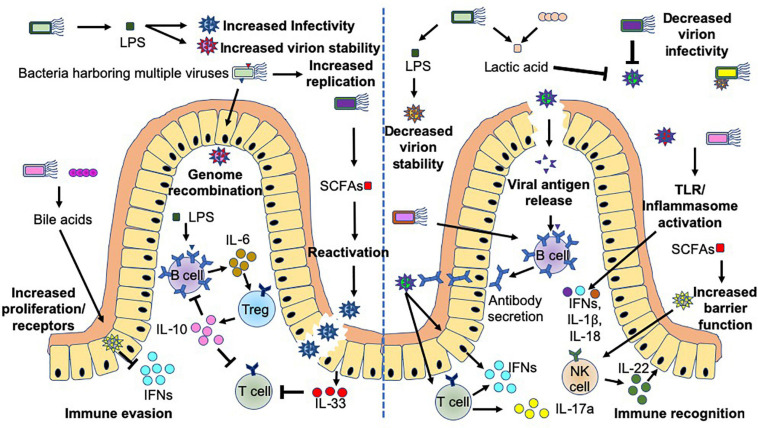
Bacteria-virus crosstalk can promote virus infection or enhance host protection. Interactions between bacteria and viruses can promote infection (left hand-side), or protection of the host (right-hand side) by multiple different mechanisms. These mechanisms focus on alterations to viral stability, replication, genetic recombination, antigen release, mucosal barrier functions and host immunity including cytokine release and antibodies. Bacteria influence viral crosstalk both directly, e.g., Lipopolysaccharide (LPS) binding to the virus or bacterial stimulation of host Toll-like receptor (TLR) or inflammasomes, and indirectly, including through the production of short chain fatty acids (SCFAs e.g., butyrate) or metabolism of bile acids. Additional abbreviations include: IFN (interferons) and IL- (Interleukin-).

Commensal bacteria exhibit both stimulatory and suppressive functions in controlling viral infection, through direct and indirect mechanisms, which can lead to harmful or beneficial outcomes for the host (Figure 4). Commensal microbiota can promote viral infections directly through the production of microbial ligands (e.g., LPS) or metabolites (e.g., Bile acids or SCFAs), which can promote: (1) target cell proliferation and upregulation of virus receptors, e.g., CD300lf (a receptor for murine norovirus) on Tuft cells (von Moltke et al., 2016; Nelson et al., 2018; Wilen et al., 2018), (2) reactivation of viruses (Gorres et al., 2014), (3) increased virion stability (Robinson C. M. et al., 2014; Berger et al., 2017), and (4) increased viral replication (Kuss et al., 2011). Importantly, norovirus infection of B cells can be promoted or prevented depending on the presence of bacteria expressing H-type histo-blood group antigens (Jones et al., 2014; Karst, 2015). In addition, enteric bacteria have been reported to enhance enteric viral (poliovirus) co-infection, which consequently can facilitate genetic recombination and enhance viral fitness (Erickson et al., 2018). Importantly, commensal bacteria can also modulate the immune response, promoting viral infection (Jude et al., 2003; Kane et al., 2011; Young et al., 2012; Uchiyama et al., 2014; Baldridge et al., 2015; Wilks et al., 2015). Two examples with different mechanisms of virus-mediated immune suppression include mouse mammary tumor virus (MMTV), which primarily infects intestinal dendritic cells, B cells and T cells (Held et al., 1993; Beutner et al., 1994) and Norovirus, which primarily infects intestinal Tuft cells (Wilen et al., 2018). In MMTV infection, MMTV in conjunction with bacterial LPS (signaling through TLR4), strongly induces proinflammatory IL-6, which in turn promotes Tregs to produce IL-10, a potent immune-suppressing cytokine, promoting viral escape from the immune system (Jude et al., 2003; Kane et al., 2011; Wilks et al., 2015). However, in norovirus infection, the presence of gut bacteria downregulates antiviral IFNλ receptor expression in the intestine and subsequently reduces intracellular downstream signaling via Stat1 and Irf3 (Baldridge et al., 2015). This should limit antiviral immunity and the survival of the virus.

On the other hand, commensal microbiota can also suppress viral infections by multiple mechanisms including: (1) directly binding to the virus, whereby the virus may be destabilized, prevented from being internalized and/or viral replication is suppressed (Botić et al., 2007; Conti et al., 2009; Mastromarino et al., 2011; Chen et al., 2016; Bandoro and Runstadler, 2017), and (2) boosting antiviral immunity and mucosal barrier integrity. This antiviral immunity includes enhancing viral antigen presentation to antigen-specific T and B cells, leading to the activation of cytotoxic CD8^+^ T cells, generation of IFN-γ-producing Th1 cells and IL-17a-secreting Th17 cells (Hensley-McBain et al., 2016), as well as synthesis of virus-specific antibodies (Ichinohe et al., 2011; Oh et al., 2014) to control and limit the infection. Furthermore, bacterial and viral ligands can activate toll-like receptors and inflammasomes, both of which promote the secretion of IFNs, IL-1β and IL-18 (Ichinohe et al., 2011; Wang et al., 2012; Oh et al., 2014) from a range of cells, while reducing IL-33 secretion (Oh et al., 2016) and enhancing IL-22 (Hensley-McBain et al., 2016) and IFN responses (Steed et al., 2017). Together, they boost antiviral immune responses and mucosal barrier integrity.

Interestingly, microbial products, such as LPS, can also both promote and suppress viral infections, which may be due to different LPS structures that have different immunostimulatory effects (Vatanen et al., 2016). This has been reported in MMTV studies, where MMTV virions bound to *E. coli* LPS had a greater ability to stimulate IL-6 production from splenocytes than *B. theta* LPS (Wilks et al., 2015). However, further studies are required to determine whether the differences in LPS from the various bacterial sources, and/or their structures, differentially modulate virus stability. Interestingly, different serotypes of *E. coli* LPS can differentially alter body temperature in rats (Dogan et al., 2000). As the peripheral circadian oscillators sense temperature changes, it is also possible that the origin of the LPS may differentially induce temperature changes *in vivo* and alter circadian rhythmicity. Furthermore, as both bacteria and TLRs oscillate, including TLR4, it is highly likely this will have an effect on viral promotion or suppression.

Bacteriophages, viruses which infect and subsequently destroy bacteria, are highly abundant and can modulate bacterial composition of the host (Hsu et al., 2019; Khan Mirzaei et al., 2020) and can prevent infection of the host by pathogenic bacteria by adhering to the mucus layer (Dubos et al., 1943; Matsuzaki et al., 2003). Importantly, bacteriophages have also been shown to be influenced by circadian rhythms (Kao et al., 2005; Liu et al., 2019). Cyanophages, which infect cyanobacteria, have been shown to exhibit diurnal rhythms in their ability to infect bacteria and in gene expression, which relates to the ability of the cyanobacteria to undergo photosynthesis (Liu et al., 2019). While this provides the first evidence of circadian modulation of phages, future studies are needed to determine whether phages in humans or mice exhibit similar changes. It will also be important to develop new technologies to fully understand the potential of these as microbial modulators and as modulators of circadian rhythms (Khan Mirzaei and Deng, 2021).

Further studies evaluating crosstalk between viruses and bacteria in circadian rhythms are urgently needed. Studies involving GF and gnotobiotic mice would be greatly insightful, so would co-infection models. Better understanding of these interactions would undoubtedly help not only to identify novel pathways for therapeutic targeting but also to better understand how therapy may inadvertently alter susceptibility to other pathogens, including opportunistic pathogens.

## Circadian Influences on Antibacterial and Antiviral Therapy

Therapies targeting the circadian clock have shown promise in controlling bacterial and viral infection. SR9009, a REV-ERB agonist, which suppresses *Bmal1*, has successfully inhibited viral entry and replication of HCV (Zhuang et al., 2019), HBV (Zhuang et al., 2021), and HIV-1 (Borrmann et al., 2020). Likewise, SR8278, a REV-ERB antagonist, which represses *Rev-erb*α and promotes *Bmal1* expression, has rendered mice more susceptible to VSV infection when infected at ZT12, compared to the more protected mice infected at ZT12 without REV-ERB antagonist treatment (Gagnidze et al., 2016). It is unknown, thus far, if Rev-erb agonists change the gut bacterial composition and function. Thus, these studies need to be conducted, and combined with studies of viral infection. While the therapies targeting circadian proteins have shown promise with respect to infection, it is important to consider broader effects. For example, Rev-erbα modulates Th17 cell development (Yu et al., 2013), and thus Rev-erbα agonists may promote cell development, while antagonists may prevent Th17 expansion. It is possible that Th17-promoting Rev-erbα agonists may subsequently result in intestinal dysbiosis in the host, due to dysregulated Th17 immune responses to gut bacteria. In addition, given the plasticity of Th17, Treg, and Th1 responses (Lee et al., 2009; Wei et al., 2009; Mukasa et al., 2010), any Rev-erbα modulation needs to be considered in all these aspects for additional immune impacts that may alter both the efficacy of treatment and susceptibility to other health problems.

The specific timing of therapeutic intervention, referred to as chronotherapy, may also play a role in modulating bacterial and viral crosstalk. In murine HSV-2 infection, time-of-day influences the survival of the infected mice, with infections at ZT6 repressing viral replication and promoting survival of the host, compared to infections at ZT18 (Matsuzawa et al., 2018). Interestingly, when these mice were infected at ZT18, a fourfold higher dose of acyclovir (administered 30 min prior to infection) was required for mice infected at ZT18, compared to those infected at ZT6 to obtain similar survival and clinical scores (100 mg/kg vs. 25 mg/kg). Therefore, the time of infection can make a substantial difference to the drug dosage needed to control the infection, which for some therapies may not be possible due to the enhanced toxicity or side effects of the drug when higher doses are used. In addition, the use of higher doses may also promote host resistance to the effective therapy. It is unclear whether antibiotics may have a similar effect on bacterial pathogens at different times-of-day. If so, administering the antibiotics at a time synchronized with the timing of optimal anti-microbial host immune responses may prove beneficial and prevent suboptimal doses of antibiotics being used, and thus reduce antibiotic resistance.

Given that the microbiota have the ability to metabolize many drugs (Zimmermann et al., 2019), and that the abundance of bacteria can oscillate, it is likely that this change in the microbiota at different times-of-day may alter the therapeutic responses that the drugs elicit. For example, certain bacterial species can enhance the success of therapy with immune checkpoint blockade in mice and cancer patients (Sivan et al., 2015; Vétizou et al., 2015; Mager et al., 2020) and gut bacteria could also enhance the responses to chemotherapy via modulating the tumor microenvironment and immune cell function (Iida et al., 2013; Viaud et al., 2013). Importantly, not only does drug-metabolism by microbiota have a local effect, but systemic consequences can occur, particularly in the liver, where hepatic drug metabolism can also be influenced by circadian oscillations (Kim and Lee, 1998; Lin et al., 2019; Wang et al., 2019). Interestingly, in the liver, Bmal1 regulates oscillations in drug metabolizing genes, as Bmal1-deficient mice showed higher levels of toxicity from metabolizing xenobiotics (Lin et al., 2019). Given that Bmal1 promotes oscillations in drug-metabolizing genes and toxicity sensitivity, and in addition, Bmal1 promotes both HBV and HCV infection in hepatocytes, some potential adverse effects of circadian therapies (i.e., Rev-erb agonists) on drug metabolism, which may result in increasing toxicity, should also be considered.

Circadian oscillations also modulate vaccine responses to bacterial and viral pathogens. The magnitude of antibody response to the trivalent inactivated influenza vaccine in humans correlated with early TLR5 expression (Oh et al., 2014). Microbial flagellin is a ligand for TLR5 (Hayashi et al., 2001) and stimulation of TLR5 boosted plasma cell numbers and antibody titers in response to the influenza vaccine (Oh et al., 2014). TLR5 was also involved in boosting antibody responses to the inactivated polio vaccine but not to adjuvanted (Tetanus-Diphtheria-pertussis) vaccine or the live-attenuated yellow fever vaccine (YF-17D). Interestingly, antibody responses to the trivalent influenza vaccine are also altered at different times-of-day, with higher titers observed in elderly individuals vaccinated in the morning compared with the afternoon (Long et al., 2016). It is unclear whether circadian oscillations in gut bacteria contribute to the alteration seen in anti-influenza antibody response; however, it is certainly possible, as TLRs oscillate and are likely to be involved in bacterial and viral ligand sensing. Further studies need to be conducted for other vaccinations and in different age groups. Given that elderly people have reduced immune responses compared to younger individuals (Gustafson et al., 2020), timing of the vaccination to boost the protective response is clinically relevant.

## Applicability of Animal Models for Human Circadian Studies

As discussed, it is clear animal models have a circadian rhythm and that both bacterial and viral organisms are able to modulate these rhythms to either promote better survival of the host or better advantage to the pathogen. While these studies have been enlightening and have some similarities with human circadian rhythm studies, it is important to consider the true implication of animal circadian studies and how these rhythms may be different to humans.

Both mice and humans have an evolutionarily preserved molecular clock and SCN, which controls the circadian rhythms in response to light; however, as mice are nocturnal and humans are diurnal their responses to light can be different. Mice can entrain to light exposure of 1 lux for a few minutes, while humans require both a high light intensity (>100 lux’s) and a longer duration (>30 min) to entrain (Daan and Pittendrigh, 1976; Khalsa et al., 2003). Thus, differences in study design related to light intensity and duration can have different effects between the species and thus the induction of circadian rhythms. Similarly, laboratory animals are generally housed in abrupt 12-h light/dark cycles, whereas humans can experience seasonal variations in light exposure (Jasser et al., 2006; Higuchi et al., 2007). Interestingly, nocturnal vs. diurnal animal species can also have a different time window and magnitude of response when serotonin is able to modulate circadian rhythms (Horikawa and Shibata, 2004; Novak and Albers, 2004; Cuesta et al., 2008). Thus, changes in response and timing need to be considered when considering translation and applicability of animal studies into human studies.

There are many other factors that may also be involved in modulating circadian differences between mice and humans. For example, changes in microbial composition between the species, as well as food type and time eaten, will also be important in determining microbial influences on circadian rhythms. Furthermore, the laboratory animals studied are often in-bred and thus, in comparison to humans, exhibit much less genetic diversity, which also may influence the applicability of murine studies to humans. However, as for other types of research that use animal models, murine studies provide a signpost for areas of investigation when considering circadian rhythms in humans. Thus, it is important for studies conducted in animal models to be confirmed in humans.

## Summary

Circadian rhythms modulate the composition and function of commensal bacteria, and host susceptibility to bacterial and viral infection. While the interactions between circadian rhythms and viral or bacterial infection have been separately studied, there is a significant knowledge gap regarding the three-way crosstalk among viruses, bacteria and circadian rhythms. Thus, the studies with co-infection (bacteria and virus) may provide important insight into the crosstalk. It is clear that circadian clock manipulation and vaccination at particular times-of-day may have important implications for boosting host protection from infection and inflammation. Thus, there is a need to better understand these interactions. As discussed earlier, not all viruses have the same time-of-day virulence to infect the host; thus, we need to consider how to target individual viruses that may alter susceptibility to infection by other viruses, opportunistic bacteria or immune-mediated diseases. By better understanding these interactions we may be able to reduce antiviral/antibiotic resistance by using lower doses of drugs, which may also result in fewer toxic side effects. Although it is difficult to pinpoint the time when an infection occurs in humans, it is possible to boost the protective immune responses at the optimal time to enhance therapeutic success, whether by vaccination or immune-modulating therapies. Finally, given the impact that circadian rhythms have on microbes and immunity, it would be important to consider the role of circadian rhythms in the design and implementation of experiments in both animal and human studies.

## Author Contributions

JAP wrote the manuscript. ACV and KB-B conducted research for the manuscript. FSW and LW edited the manuscript. All authors contributed to the article and approved the submitted version.

## Conflict of Interest

The authors declare that the research was conducted in the absence of any commercial or financial relationships that could be construed as a potential conflict of interest.

## Publisher’s Note

All claims expressed in this article are solely those of the authors and do not necessarily represent those of their affiliated organizations, or those of the publisher, the editors and the reviewers. Any product that may be evaluated in this article, or claim that may be made by its manufacturer, is not guaranteed or endorsed by the publisher.
